# Cyclic Guanosine Monophosphate–Adenosine Monophosphate Synthase (cGAS), a Multifaceted Platform of Intracellular DNA Sensing

**DOI:** 10.3389/fimmu.2021.637399

**Published:** 2021-02-23

**Authors:** Eloi R. Verrier, Christelle Langevin

**Affiliations:** ^1^Université de Strasbourg, Inserm, Institut de Recherche sur les Maladies Virales et Hépatiques UMR_S1110, Strasbourg, France; ^2^Université Paris-Saclay, INRAE, IERP UE0907, Jouy-en-Josas, France

**Keywords:** CGAS, STING, innate immunity, evolution, *in vivo* models

## Abstract

Innate immune pathways are the first line of cellular defense against pathogen infections ranging from bacteria to Metazoa. These pathways are activated following the recognition of pathogen associated molecular patterns (PAMPs) by membrane and cytosolic pattern recognition receptors. In addition, some of these cellular sensors can also recognize endogenous danger-associated molecular patterns (DAMPs) arising from damaged or dying cells and triggering innate immune responses. Among the cytosolic nucleic acid sensors, the cyclic guanosine monophosphate–adenosine monophosphate (cGAMP) synthase (cGAS) plays an essential role in the activation of the type I interferon (IFNs) response and the production of pro-inflammatory cytokines. Indeed, upon nucleic acid binding, cGAS synthesizes cGAMP, a second messenger mediating the activation of the STING signaling pathway. The functional conservation of the cGAS-STING pathway during evolution highlights its importance in host cellular surveillance against pathogen infections. Apart from their functions in immunity, cGAS and STING also play major roles in nuclear functions and tumor development. Therefore, cGAS-STING is now considered as an attractive target to identify novel biomarkers and design therapeutics for auto-inflammatory and autoimmune disorders as well as infectious diseases and cancer. Here, we review the current knowledge about the structure of cGAS and the evolution from bacteria to Metazoa and present its main functions in defense against pathogens and cancer, in connection with STING. The advantages and limitations of *in vivo* models relevant for studying the cGAS-STING pathway will be discussed for the notion of species specificity and in the context of their integration into therapeutic screening assays targeting cGAG and/or STING.

## Introduction

Type I interferons (IFNs) can be secreted by a wide range of immune and non-immune cells in response to various biological stimuli [danger associated molecular patterns (DAMPs) and pathogen associated molecular patterns (PAMPs)] that activate nuclear, cytosolic, or membrane-anchored nucleic acid sensors (1, 2). Discovery and characterization of these specialized receptors, which trigger innate immune responses, started in early 2000 with the description of lipopolysaccharides (LPS) and CpG sensing by TLR4 and TLR9, respectively (3, 4). Since then, extensive investigations have been conducted to identify cytosolic DNA receptors classified as DNA sensor based on DNA binding activity and activation of innate immune responses (5). Recent evidence highlighted their diversity in terms of structure/function, patterns of expression, and signaling pathway (5). This raises important questions on the existence of ligand specificity, the impact of the tissue environment, and the orchestration of overlapping DNA signaling pathways (6). Indeed, numerous DNA sensors have been identified, which belong to PYHIN proteins (HIN200 domain-containing proteins) such as interferon gamma-inducible protein 16 (IFI16) and absent in melanoma 2 (AIM2); to DExH-box helicases (DHX9 and DHX36) or DEAD-box helicase family (DDX41) and to proteins involved in responses to DNA damage (MRE11, or Rad50 and DNA-PK). In addition, DNA-dependent activator of IFN regulatory factors (DAI), RNA polymerase III (RNA pol III), and LRR binding FLII interacting protein 1 (LRRFIP1) were also involved in DNA sensing and type I IFN response [for reviews (5, 7)]. However, the depletion of some of these sensors (DDX41 or DAI) in mouse or cellular models does not always correlate with an impact on DNA-stimulated type I IFN response, which highlights the need for further studies (5). Finally, the cyclic guanosine monophosphate–adenosine monophosphate (cGAMP) synthase (cGAS) has emerged as central to the mounting of nucleic acid-dependent IFN responses *in vivo* (8). It is involved in the detection of a wide range of cytosolic DNA ligands from self and non-self origins. Association of human cGAS (also known as C6orf150 encoded by *MB21D1*) with dsDNA catalyzes the production of cyclic cGAMP. Of note, ssDNA (9) and RNA:DNA hybrids (10) have been shown to activate cGAS leading to cGAMP production. This second messenger triggers the activation of innate immune responses by binding to the adaptor protein STING (also known as MITA, ERIS, or MPYS, encoded by *TMEM173*). STING recruits the TANK-binding kinase 1 (TBK1) and the inhibitor of nuclear factor kappa-B *kinase* subunit epsilon (IKKε) and activates the IRF3 and the nuclear factor kappa (NF-κB)-light-chain-enhancer of activated B cells transcription factors (11, 12). The exact location of STING/TBK1 interaction is still in debate. Induction of the cGAS-STING pathway culminates in the synthesis of type I IFN and pro-inflammatory cytokines (13, 14). Notably, activation of cGAS-STING pathway leads to the establishment of an IFN-based and IFN-independent innate immune response (15–17).

Fine-tuning of the cGAS-STING pathway is necessary to initialize and resolve inflammatory processes, maintain tissue homeostasis, fight against pathogen infections (i.e., bacteria, viruses, and parasites), and modulate the immunity of the tumor microenvironment (toward tumor suppression or tumor and metastasis development in a different context) (18). Therefore, the role of cGAS-STING in auto-inflammatory and autoimmune diseases has been established leading to a chronic activation of the IFN pathway, which can be detrimental (16). This includes inflammatory syndromes such as STING-associated vasculopathy with onset in infancy (SAVI), Aicardi–Goutières syndrome (AGS), and familial chilblain lupus (19–23), but also cGAS related genetic disorders such as TREX1 associated lupus-like autoimmune disorder (24) or Bloom syndrome (19). Systemic inflammation triggers complex pathological phenotypes with multi-organ damages. Although ubiquitously expressed, a growing body of evidence demonstrates the existence of cell- and tissue-related variability in the expression pattern of cGAS-STING (25) as described for IFN and interferon-stimulated genes (ISG) from mammals to zebrafish (26–29). cGAS and STING expressions are IFN-inducible and are involved in the regulation of the type I IFN feedback loops (30). According to the tissue distribution described in the Human Protein Atlas (http://www.proteinatlas.org), *MB21D1* and cGAS protein are ubiquitously expressed with particularly high expression in epithelial cell types in the genital tract or the lungs as well as in hematopoietic cells and dendritic cells, with *TMEM173*/STING presenting a quite comparable distribution pattern (31, 32). In contrast, primary human hepatocytes express low levels of cGAS and STING (33, 34). One putative explanation would be that low cGAS and STING expression would avoid overactivation of this pathway during hepatocyte renewal, which leads to DNA accumulation in the cytoplasm (34, 35). Recent evidence suggests a complex interplay between cGAS and STING in the liver involving multiple cell types, as it has been suggested that cGAMP could be transferred from hepatocytes to liver macrophages (expressing high levels of STING) through gap junctions (36, 37).

In addition, the cGAS-STING pathway has been involved in cancer immunity and the development of immunotherapies. The extensive works carried out to understand the correlation between expression of cGAS/STING and cancer will not be discussed in this review but recently presented in (18, 38).

A better understanding of the cGAS-STING multifaceted platform is required to improve our knowledge of the orchestration of innate immune responses mediated by diverse nucleic acid sensors, activated by self and non-self motifs in a tissue-specific manner. Animal models are critical to predict physiologically relevant functions of the cGAS-STING pathway *in vivo* taking into account the cell and tissue environments in different physiological states (16). Despite an important evolutionary conservation of the cGAS-STING functions in innate immunity, recent data have highlighted certain species specificities, which must be considered when using biomedical models for the identification of biomarkers or therapeutic screening for human health (8, 39, 40). In this review, we depict the evolution and the broad biological functions of the cGAS-STING DNA sensing platform in pathogen recognition, immune activation, and cancer development, as well as its potential for the development of novel therapeutic strategies.

## Origin and Evolution of the Molecular Mechanisms of the Nucleic Acids—cGAS-Sting Interactions

**Figure 1:**
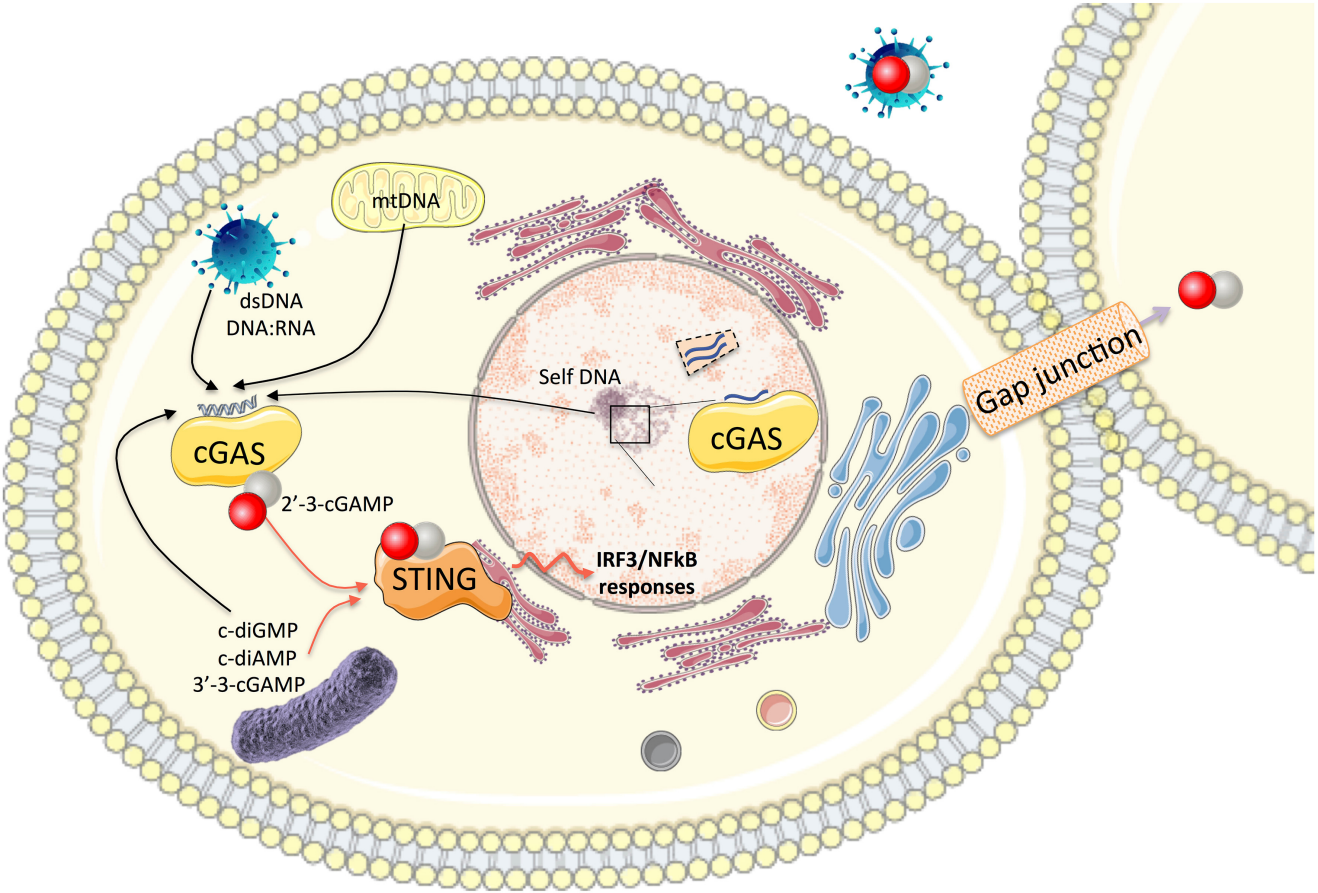
In the cytosol, the association of cGAS with self and non-self cytosolic nucleic acid substrates catalyzes the production of 2′-3′-cGAMP. This second messenger binds to the adaptor protein STING and activates the IRF3 and NF-κB transcription factors for the synthesis of type I IFN and pro-inflammatory cytokines. cGAMP can be transported to neighboring cells through gap junctions or integrated into viral particles, leading to autocrine or paracrine activation of STING. Bacterial dinucleotides (c-diGMP, cdiAMP, and 3′-3′-cGAMP) are also ligands of STING. cGAS also impacts nuclear functions (impairment of DNA repair, genome destabilization, and synthesis of micronuclei). Nuclear cGAS discriminates between self and non-self DNA by binding to chromatin (preventing its activation) or interaction with nuclear proteins such as NONO in response to viral infection to trigger innate immune responses.

cGAS is composed of a flexible and poorly conserved N-terminal domain and a highly conserved C-terminal catalytic domain composed of nucleotidyltransferase (NTase) core and Mab21 domains [reviewed in (41) and (42)]. The sequence-independent DNA sensing activity contained a positively charged surface and a zinc-ribbon domain. Upon activation, the cGAS dimer exposes a catalytic site formed by a caged tertiary structure composed of typical alpha helices (ligand-binding surface) and the nucleotidyltransferase core domain. Binding of mislocated or infectious cytosolic DNA to cGAS catalyzes the production of 2′-5′/3′-5′ cyclic GMP–AMP, the 2′3′-cGAMP second messenger (Figure 1) (43). pppGp(2′-5′)G or 2′,3′-c-di-GMP were also detected as minor products in the absence of ATP (43). Structural homologs of human cGAS have been identified in animals and bacteria. In eukaryotes, it includes metazoans and human proteins such as the antiviral oligo adenylate synthase 1 (OAS1), which produces 2′,5′-oligoadenylate (2–5A) upon sensing of the cytosolic double-stranded RNA. The 2–5A ligand further activates the endoribonuclease RNase L, leading to RNA degradation. In bacteria, the dinucleotide cyclase DnCV of *Vibrio cholerae* is considered to be a founding member of a large family of cGAS homologs, which synthesizes 3′-3′-cGAMPs as well as trinucleotides and oligonucleotides in absence of activation (44). Overall, the structure of the unique catalytic site, which ensures nucleotidyltransferase and dinucleotide cyclase activities in a sequential fashion (43), is an important conserved feature despite low sequence homologies. Is oligomerization necessary for activation? The answer is not really clear, although it clearly contributes to regulate the enzymatic function (42).

Functional characterizations of the nucleotide synthesized by the cGAS-related proteins have been conducted in eukaryotes and bacteria. While cGAMP second messenger triggers innate immunity in mammalian cells by binding to STING, bacterial nucleotides can be recognized not only by phospholipases and riboswitches but also by nucleases, proteases, or pore-forming effectors. Moreover, microbial DNA, cyclic dinucleotides, and host DNA (either mitochondrial- or mislocated self-DNA) were identified as STING ligands capable of inducing the activation of the innate immune response in Metazoa (Figure 1) (45). A recent study elegantly dissecting the STING-dependent pathway characterized functional STING homologs in bacteria and demonstrated the conservation of a prokaryotic cGAS-STING-like pathway playing a role in the antiviral defense against bacteriophages (46). STING is an ER protein composed of four transmembrane domains and a cytosolic domain formed by an alpha helix, a cyclic dinucleotide binding domain (CBD), and a C- terminal tail (CTT) carrying the binding sites for TBK1 and IRF3. Phylogenetic comparisons of invertebrate and vertebrate STING versions highlighted that ray-finned fish acquired a signaling module at the extremity of the CTT domain mediating TRAF6 interaction and promoting the transcription of NfκB responsive elements (47), while the sea anemone (*Nematostella vectensis*) lacks the CTT region (11). Functional characterization of STING from diverse animal lineages showed that purified CBD domains from vertebrates could bind 2′-3′-cGAMP. Recognition of 3′,3′-dinucleotides was restricted to mammalian STING orthologs. STING alleles from insects showed no interaction with any of the cyclic dinucleotide (CDN) substrates tested in the assay (11). STING homologs identified in invertebrates (Annelida, Mollusca, and Cnidaria phylum) have conserved the ability to bind 3′-3′-cyclic dinucleotides and 2′-3′-cGAMP despite low sequence identity outside the key residues conserved in divergent STING homologs and implicated in the CDN recognition. Thus, the recognition of the endogenous 2′-3′-cGAMP ligand is a conserved hallmark supported by the unique conformation of the CBD domain of STING receptor in complex with 2′-3′-cGAMP. Comparative studies of human and sea anemone 2′-3′-cGAMP—STING structures showed that they maintained this conservation despite low sequence identity (11). Dinucleotide sensing triggers the activation of type I IFN responses mediated by human STING expression in contrast to the absence of stimulation monitored after the sea anemone STING expression. This has been correlated with the absence of the CTT domain in the sea anemone STING protein since fusion with the human CTT domain is sufficient to restore activation of IFN in response to 2′-3′-cGAMP exposure (11). The STING signaling pathways (IRF3/NFκB) therefore depends on CDN ligand selectivity and conformation of the CBD domain related to the orientation of the b strand lid domain (on the top of the ligand-binding pocket) and of the CTT (regulating the transition from activated to inactivated state) (48). Molecular dynamics simulations comparing human and mouse STING conformations (opened-inactive or closed-active) have been instrumental in describing the species-specificity of STING in an Apo conformation or upon binding to the DMXAA agonist (49). This species-specificity must be taken into account when considering the applicability of the results obtained for STING agonists using mouse models before they enter into clinical trials (50). At the molecular level, the modulation of STING functions occurs through palmitoylation (51), protein multimerization, and translocation from ER to Golgi (52, 53) for the recruitment of downstream signaling partners (13). Auto-inflammatory syndrome related to mutations in coatomer protein subunit α, COPA (that mediates Golgi to ER transport) was recently attributed to the retention of STING dimers in the Golgi in the absence of cGAMP stimulation. This triggers an enhanced and unregulated type I IFN activation similar to STING mutants of the SAVI-associated syndrome localized in the Golgi in the absence of stimulation (53–56). The link between STING multimerization and its activation process was recently re-evaluated by Ergun et al. using structural biology and biochemistry. They showed that the nature of STING polymers (inter-dimer crosslinks) depends on the ligand. Polymers are blocked by the CTT domain and are formed in the RE prior to trafficking to the Golgi (57). In line with this observation, R284S STING mutants (SAVI-associated syndrome) were shown to generate constitutive polymers related to chronic STING activation (57).

However, the molecular mechanisms involved in the control of the cGAS-STING pathway are still poorly understood. The hydrolysis of 2′-3′-cGAMP messenger by the ecto-nucleotide pyrophosphatase/phosphodiesterase (ENPP1) constitutes one of these mechanisms (58). Since its discovery, this extracellular enzyme has aroused great interest because of its strong therapeutic potential (59) as inhibitors of ENPP1 could help potentiate cGAS-STING signaling (60). cGAS-STING pathway is also modulated by the epidermal growth factor receptor (EGFR) (61). EGFR is required for the phosphorylation of STING in the ER, leading to its endosomal translocation to activate IRF3 (61). In addition, the lysyl-tRNA synthetase has recently been identified as a potent modulator of the STING-dependent IFN pathway in a two-step mechanism (45). First, it competes with cGAS for the binding of cytosolic nucleic acid ligand, thus impeding the production of cGAMP. Second, its activation leads to the production of diadenosine tetraphosphate (Ap_4_A), an endogenous antagonist of STING. Interestingly, the lysyl-tRNA synthetase- Ap_4_A axis modulates the IFN pathway *in vitro* and *in vivo* in zebrafish larvae, suggesting an ancestral mode of regulation of cGAS-STING functions conserved across vertebrates (45).

The cGAS family has several features in common with the STING family conserved during the metazoan evolution (42, 46, 48), as they are present early in several simple organisms (42, 46, 48) but were subsequently lost in nematodes and flatworms (11). The study of the molecular evolution of cGAS and STING has shown the important conservation of the catalytic site (cGAS) and cyclic dinucleotide binding domain (STING) despite low sequence homologies (11, 42, 46, 48). It has also revealed the emergence of the zinc-ribbon domain and the N-terminal fragment of cGAS that ensure its ligand specificity and stability (42) as well as the CTT domain of STING carrying the binding sites of signaling molecules (47). The functional conservation of the cGAS-STING pathway highlights its central role in the cellular response to DNA sensing. In the next paragraph, we will present a concise description of the broad cellular functions of human cGAS.

## cGAS: A Main Actor of Cellular Response to DNA and RNA Viruses

As described above, cGAS is considered as the main sensor of DNA viruses in the cytoplasm of infected eukaryotic cells. Of note, the bacterial homolog of cGAS belongs to a four-gene operon mediating antiviral defense against a broad variety of phage infection. This relied on cGAMP production and phospholipase activation leading to cell death (62). Recently, analogs of eukaryotic STING presenting a comparable mechanism of cGAMP-mediated activation were characterized, suggesting the conservation of this antiviral system from bacteria to metazoans (46). In this paragraph, we mainly present the interaction between human cGAS and a broad range of DNA viruses. Of note, such viruses usually replicate in the nucleus of infected cells, and their genome is often protected within the capsid in the cytoplasm during infection, limiting their detection by cytosolic sensors.

Initially, cGAS was described as a cGAMP synthase required for IRF3 dimerization following infection of murine fibroblast cells with herpes simplex virus type-1 (HSV-1), a DNA herpesvirus previously known to induce the expression of IFNs through the STING-IRF3 axis (63). The importance of cGAS in mounting the antiviral response against HSV-1 and survival to infection has been further determined *in vivo* (8). Interestingly, it has been shown that HSV-1 capsid was ubiquitinated upon infection of dendritic cells, leading to its degradation by the proteasome and the release of viral DNA in the cytoplasm, triggering its detection by DNA sensors (64). The DNA genome of poxviruses is also recognized by cGAS to induce the innate immune response. Indeed, the knockdown of cGAS inhibits the induction of IFNβ following vaccinia virus (VACV) infection in THP1 cells (63). Notably, cGAS-mediated detection of VACV leads to the production of cGAMP that could be efficiently transferred to bystander cells, triggering the activation of a STING-dependent antiviral immunity in non-infected cells (65). Different models of cell-to-cell transfer of cGAMP have been proposed occurring through extracellular vesicles such as exosomes (66), gap-junctions (67, 68), and incorporation into enveloped viruses (69) in addition to the recently described cGAMP transporters (70–72). Importantly, cGAS was rapidly described as a main sensor of HIV and other retroviruses (73). In the absence of cGAS, HIV, murine leukemia virus, and simian immunodeficiency viral infections do not elicit antiviral response (73). cGAS notably recognizes specific Y-form DNA motifs from HIV-1 in the cytoplasm of infected macrophages (9), and possibly the RNA:DNA hybrids accumulating in the cytoplasm of retrovirus-infected cells (10). Interestingly, by studying the interaction between HIV and cGAS, two independent teams demonstrated the ability of HIV to encapsidate cGAMP within neosynthesized virions, thus leading to paracrine activation of a STING-dependent IFN response in newly infected cells (66, 69). The structure of the capsid is an essential determinant of cGAS-mediated sensing of the cDNA of HIV in dendritic cells, which does not require genome integration (74). Recently, NONO was described as a major actor of HIV capsid detection in the nucleus. NONO directly interacts with HIV capsid in the nucleus of dendritic cells and is required for the presence of cGAS in the nucleus and cGAS-mediated detection of HIV DNA (75). Hence, the detection of HIV capsid by NONO enables the sensing of HIV DNA by the nuclear cGAS, suggesting a novel role of cGAS in the activation of innate immunity in the nucleus and a cellular strategy to distinguish self-DNA from viral DNA in the nucleus of infected cells (75). By redefining cGAS localization patterns, recent studies corroborated this observation, describing cGAS activity in the nucleus [reviewed in (76)], for instance in the context of DNA damage, raising questions regarding the interaction between cGAS and self-genomic DNA (77). Several groups recently demonstrated the importance of extensive binding of cGAS to chromatin in the prevention of cGAS oligomerization and activation, proposing the first clear mechanisms allowing cGAS to discriminate self from non-self-DNA in the nucleus (78–80). These observations were further supported by the role of nuclear histones in suppressing the cGAS mediated immunogenicity of self-DNA (81–83). In line with the importance of cGAS sequestration by histones in limiting its antiviral activity, it was recently described that histone deacetylase 4 restricts DNA viruses such as HSV or VACV through the induction of IFN response (84). Another step to the regulation of cGAS involves the cellular protein barrier-to-autointegration factor 1 (BAF), competing to bind to self-DNA in the context of a breakdown of the nuclear envelope integrity (85). These recent data have important conceptual implications in the interaction between cGAS, cellular components, and viral DNA in the nucleus, even though no direct interaction has been observed apart for HIV capsid so far. In this context, several lines of evidence suggest that the DNA genome of hepatitis B virus (HBV) stimulates cGAS activity and triggers the activation of the cGAS-STING pathway when transfected into hepatocyte-derived cells (33, 86). However, no induction of innate immune pathways is detected upon viral infection (33, 86). The “stealth” pattern of this peculiar virus was initially attributed, in addition to the absence HBV RNAs sensing, to the protection of the genome within the capsid during its transport to the nucleus (87). The recent data confirming the presence of cGAS in the nucleus raise the question of its ability to interact or not with the specific forms of HBV DNA in the nucleus, including the minichromosomal structure covalently closed circular DNA (cccDNA) that serves as a template for the transcription of viral RNAs (88). In this context, the low expression of cGAS and STING in the hepatocytes may also explain the absence of quantifiable induction of IFN response upon infection (34).

Mirroring the central role of cGAS in the innate antiviral response, a high diversity of mechanisms of viral evasion from the cGAS-STING pathway has been described, some of them directly interacting with cGAS [reviewed in (89)], such as KHSV ORF52 that inhibits its enzymatic activity by blocking cGAS DNA binding (90). Virus-induced degradation of cGAMP has also been investigated by performing a biochemical screening of 23 different mammalian poxviruses. It allowed the identification of viral nucleases classified as poxvirus immune nucleases (poxins) (91) for which homologs have been described in insect viruses and bacteriophages. These proteins represent now a broad family of 369 members identified in viral and animal genomes, potent modulators of the cGAS-STING pathway (92). Of note, viruses that theoretically do not trigger the activation of cGAS are also able to inhibit its activity or expression. For instance, HBV infection leads to a decrease in cGAS and STING expression in infected hepatocytes-derived cells and infected liver, both *in vitro* and *in vivo* (86). Importantly, numerous members of the RNA virus family *Flaviviridae* exhibit an impressive variety of mechanisms regulating the cGAS-STING pathway [reviewed in (93)], such as dengue virus (DENV) protease cofactor NS2B that triggers cGAS degradation in an autophagy-dependent manner (94) and Zika virus NS1 that prevents caspase-1 degradation, leading to cGAS cleavage and modulation of type I IFN signaling (95). At the current stage of our knowledge, no typical genomic or intermediary structures from Flaviviruses are susceptible to be detected by cGAS, raising the question of RNA virus evolution leading to the counteraction of this innate immune pathway in the absence of direct sensing (93). Of note, independently from cGAS, influenza A viral particles have been shown to directly interact the STING through its fusion peptide, thus stimulating IFN response (96). Regarding cGAS, Schoggins et al. observed that cGAS activation led to the development of a broad antiviral response, targeting both RNA and DNA viruses (39). The same study elegantly demonstrated that cGAS-depleted mice were much more susceptible to West Nile virus (WNV) infection, an RNA virus whose detection by the innate immune system does not rely on cGAS (39). These observations suggest a central and broad function for cGAS in the establishment of the innate antiviral response, even in absence of the direct sensing of viral genomic structures. It raises the question of an unknown ligand or crosstalk of signaling pathways that triggers the activation of cGAS to establish a basal antiviral state in the cells, with the ability to control virus infection. In this context, many RNA viruses interact with the cellular DNA repair machinery, leading to DNA damage that may serve as a cGAS ligand upon infection [reviewed in (97)]. Notably, viral oncogenes, such as E7 from the human papillomavirus (HPV), E1A from the adenovirus, and the simian virus 40 (SV40) large T antigen have been shown to modulate the cGAS/STING pathway (98, 99). In the specific case of Flaviviruses, it has been proposed that leaking mitochondrial DNA coming from damaged mitochondria upon DENV infection may trigger cGAS activation in the cytoplasm of infected cells (93). A more comprehensive knowledge of cGAS ligands is still required to understand the complex interaction between cGAS and the diversity of virus infections.

## cGAS Interaction with Metazoan Parasites

As intracellular pathogens, several multicellular parasites also trigger the cGAS-STING pathway following the sensing of DNA structures, such as *Toxoplasma gondii*, one of the most common parasites in developed countries and responsible for toxoplasmosis (100), *Trypanosoma cruzi*, a member of euglenoids causing Chagas disease in humans, or *Leishmania* [reviewed in (17)]. In the same vein, genomic DNA from *Plasmodium falciparum*, the causative agent of malaria, is detected by cGAS following infection, leading to type I IFN production and systemic inflammation, with hemozoin, the product from blood digestion by *P. falciparum* playing a key role in the delivery of genomic DNA in the cytosol (101). The importance of cGAS in the control of *P. falciparum* infection was confirmed *in vivo*, as cGAS-depleted mice showed a higher susceptibility to parasitic infection (102). Interestingly, computational analysis from *in silico* screening as well as IFN inhibition assay in a mouse model of AGS syndrome suggested that several antimalarial drugs, such as hydroxychloroquine or X6, could interact with cGAS and inhibit DNA-cGAS interactions, blocking IFN response (103, 104). However, the involvement of this mode of action in the control of *P. falciparum* infection remains to be determined (17).

## cGAS and Bacterial DNA: From Host Defense to Interaction with Microbiota

cGAS has been also shown to be an important sensor of intracellular bacteria. Three groups simultaneously described the involvement of cGAS in the detection of microbial DNA from *Mycobacterium tuberculosis*, the causative agent of tuberculosis. cGAS and *M. tuberculosis* are notably colocalized in the human tissue from patients with tuberculosis, and cGAS depleted mice are more susceptible to bacterial infection (105). Infection of macrophages revealed a STING-dependent activation of antimicrobial response following direct binding of cytosolic DNA to cGAS, leading to an autophagy-driven elimination of *M. tuberculosis* (106). Notably, *M. tuberculosis* strains isolated from patients with severe tuberculosis do not induce a robust induction of cytokines upon infection of macrophages, including weak induction levels of interleukin-1β (IL-1β) associated with evasion from cGAS sensing (107). Similarly, both cGAS and STING are required for INFβ production following infection of multiple cell types by *Chlamydia trachomatis*, a Gram-negative bacterium mainly causing disease of the genital tract (108). Interestingly, *C. trachomatis* inclusion protein CpoS inhibits the cGAS-STING pathway by targeting STING and limiting apoptosis of the infected cells (109). *Listeria monocytogenes*, a Gram-positive bacterium replicating in myeloid cells, induced IFNβ expression through both IFI16 and cGAS detection upon infection (110). Interestingly, DNA from *L. monocytogenes* can be transferred from infected cells to neighboring naïve cells through extracellular vesicles, leading to the paracrine activation of the cGAS-STING pathway. This was also observed upon infection of both *Francisella tularensis* and *Legionella pneumophila*, suggesting a general pathway of innate immune activation following bacterial infection (111). Independent from the microbial-induced IFN response, STING activation and binding to ITPR1 upon infection play a key role in coagulation and mortality associated with sepsis in animal models infected by *Escherichia coli* or *Streptococcus pneumoniae* through Gasdermin D activation and F3 release (112). In the same vein, the upregulation of STING pathway is also associated with sepsis-associated mortality in patients (112).

In contrast to the above examples for pathogenic bacteria, cGAS-STING also interacts with commensal bacteria and constitutes important regulators of host-commensal microbiota interactions, which contribute to maintaining gut homeostasis through modulation of the host inflammatory response and function of the gut barrier. Indeed, in this tissue environment, the sensing of genomic DNA from invading pathogens (mediated by cGAS) and of cyclic dinucleotides generated by commensal bacteria (mediated by STING) should be tightly regulated to avoid an exacerbated inflammatory response and preserve intestinal integrity. Studying the role of STING in sepsis pathophysiology in a pilot experiment, Hu et al. sampled human intestine biopsies from patients with sepsis in comparison to healthy control biopsies. Histological analyses have correlated the level of STING expression with tissue injury, apoptosis, and intestinal inflammation (113). This was further investigated in a mouse model of sepsis with STING knock-out (KO) animals, which confirms that the control of the STING-mediated intestinal inflammation allows an improvement of intestinal barrier function and tissue histopathology (113). These results are reminiscent of the elements of clinical diagnosis of human patients with abdominal sepsis and the observations made from other rodent models. In steady-state, Sting^−/−^ KO mice models showed defective intestinal homeostasis functions (altered pattern of villi, decreased number of goblet cells, and mucus vesicles per villi as well as lower levels of secreted IgA) and an immature intestinal immunity similar to the phenotype previously described for germ-free mouse models (114). The composition of the microbiota is also impacted by STING since KO mice presented an increase in pro-inflammatory bacteria (114). Upon intestinal injury (dextran sodium sulfate-induced colitis, T-cell-induced colitis, and enteric *Salmonella typhimurium* infection), STING KO mice develop more severe signs of morbidity and an impaired pro-inflammatory immune response compared to wild-type (WT) mice (114). Therefore, regulation of STING pathway is essential to maintain gut homoeostasis and to activate host innate immune responses.

The influence of cGAS is much less understood but does not seem to directly impact the composition of microbiota or the maintenance of the intestinal homeostasis in a mouse model of dextran sodium sulfate-induced colitis (114). However, cGAS has recently been described as a scaffolding protein, which facilitated the internalization of extracellular cyclic dinucleotides (from self and non-self origin) prior to its binding, which precedes the formation of STING signalosomes and its activation (115).

Hence, the crosstalk between cGAS-STING signaling and pathways activated by an increasing diversity of innate immune sensors, complicates the understanding of host-commensal microbiota interactions and the regulation of intestinal homeostasis (116).

## Analyzing cGAS-Sting Functions *in vivo*: Similarities and Divergences in Model Organisms

Comparative analyses of cGAS-STING pathways in various model organisms have shown the conservation of the activating functions of the type I IFN response despite the diversification of the molecular mechanisms during evolution (6). Ectopic expression of genes encoding vertebrate *Sting* in human cells was used to screen their ability to induce NFκB and IRF3 responding elements. While mammalian STING induced a stronger IRF3 response than NFκB, expression of STING from fish species results in a higher NFκB stimulation compared to IRF3. This phenotype was dependent on the expression of a fish-specific minimal motif in the CTT domain of STING that recruits TRAF6 and promotes NFκB activation (47). Further studies will be needed to demonstrate the role of the STING-TRAF6-NFκB signaling axis in the innate immune responses observed *in vivo*. Interestingly, the activation of the STING-TRAF6-NFκB axis was also reported in different human cell types in response to DNA damage (117). Two studies performed in zebrafish larvae demonstrated the role of zebrafish STING in inducing the expression of type I IFN genes during infection with HSV-1 (118) or detection of hypomethylated DNA (119). In contrast to mammalian species, zebrafish cGAS is dispensable for HSV-1 DNA sensing, which occurs through the alternative DNA sensors DHX9 and DDX41 (118). The recent discovery of another functional cGAS isoform in the zebrafish genome prompted a re-examination of the role of cGAS in the sensing of HSV-1 (120). The possible involvement of pangolins during the emergence of the coronavirus disease-2019 (COVID-19) pandemic puts forward the question of the mechanisms of detection of cytosolic nucleic acid in this species, which has been shown to be infected by viruses closely related to severe acute respiratory syndrome coronavirus 2 (SARS-CoV-2). Comparative genomics of phylogenetical analyses revealed that cGAS and STING have been inactivated in pangolin species by mutations and premature stop codons. This points again to the importance of combining various animal models for the study of innate immune mechanisms and the characterization of alternative mechanisms of nucleic acid sensing (121). In the same vein, as another potential reservoir of SARS-CoV-2-related viruses, an altered IFN response due to a key mutation in the bat version of STING was recently reported (122).

The cGAS-STING pathway was considered as non-dispensable for the detection of DNA viruses *in vivo* (8, 123). However, recent work demonstrated the existence of a STING-independent DNA immune response occurring through the detection of cytosolic dsDNA by the DNA-PK DNA repair pathway. This DNA-PK-dependent IFN production appears to be limited to human cells as it could not be demonstrated in murine cells (124). Alternative *in vivo* models thus contribute to reassessing the impact of other sensing pathways and of the specificities of the species considered (6). Other illustrations of species specificities arise from infectious models for HSV-1 and Zika virus infections. To counter cellular antiviral responses and ensure their replication in the host organism, viruses have developed evasion mechanisms targeting IFN responses and cGAS-STING pathways (89). RNA and DNA viruses inhibit cGAS or STING by inducing their degradation or blocking their interactions with signaling proteins such as TBK1 (89). Interestingly, these processes present cell-(89) and species-specificities (125). Indeed, STING can promote HSV-1 infection in HEp-2 or HeLa cells (in an ICP0 dependent manner), while it is involved in the antiviral response described in human embryonic lung cells (126). In another study, host susceptibility to Zika virus has been investigated in fibroblasts obtained from human, primate, and murine cells. This comparative analysis showed that the murine fibroblasts are partially resistant to viral infection in contrast to the human and primate cells based on a STING-dependent restriction mechanism. The authors further demonstrate that human STING is targeted for degradation by the NS2B3 viral proteases of four distinct flaviviruses (ZIKV, DENV, WNV virus, and Japanese encephalitis virus) in contrast to murine STING, which does not share the protease cleavage site (125). However, infection of *Sting* KO mice does not recapitulate the *in vitro* observations as the mice have become hypersensitive to Zika infection. This highlights the complexity of co-existing antiviral mechanisms, which co-orchestrate the innate immune response in a cell-and species-specific manner.

## Discussion and Perspectives: cGAS-Sting as a Targetable Pathway in Therapy

In addition to its role in anti-pathogenic surveillance and response, accumulating evidence suggests a key role for cGAS in immune activation in cancer cells. Numerous studies reported an antitumor role for the cGAS-STING pathway. This topic has been extensively treated elsewhere (18, 38) and will not be developed in this review. The central role of the cGAS-STING pathway in various human pathologies such as cancer, infections, autoimmune diseases, and inflammatory diseases has prompted the search for therapeutics targeting the cGAS-STING-TBK1 axis (127). The modulation of immune responses remains one of the approaches considered in the treatment of these diseases through the improvement and/or refinement of existing strategies. Indeed, anti-inflammatory [systemic lupus erythematosis (SLE), STING-associated vasculopathy with onset in infancy (SAVI), and Copa syndrome (COPA)], anti-viral (hepatitis and HIV) and anti-tumor treatments target type I IFN signaling. However, significant side effects have been reported resulting from the difficulty in controlling the extent and duration of the IFN response *in vivo* (127). Therefore, extensive studies are being conducted to identify alternative treatments, some of them focusing on agonists and antagonists of the cGAS-STING complex, using *in silico* and high-throughput screening approaches (13, 127, 128). Other approaches target modifying enzymes involved in the synthesis of STING ligands and/or the post-translational modifications of cGAS and STING (129). In addition, targeted approaches are being developed based on the modulators of the cGAS-STING pathway such as the immunosuppressor MYSM1, which may be considered as a therapeutic target for inflammatory and autoimmune diseases (130).

Initial lead candidates are further characterized *in viv*o for stability, pharmacological properties, pharmacodynamics, and toxicity. Nanocarriers (such as nanoparticles, liposomes, or viral particles) have improved the efficacy and delivery of molecules targeting cGAS-STING, used in the treatment of solid tumors, lymphomas or to potentiate influenza vaccine response (131–133). In addition, using a mass spectrometry-based ligand screening technique, Siu et al. successfully generated STING antagonist molecules based on their compatibility with oral administration and efficacy to stabilize human STING dimer in an inactive conformation (134). The development of physiologically relevant biomedical models of cGAS-STING related pathologies is thus essential to validate the efficacy of therapeutic candidates but above all to predict the potential side effects linked to the modulation of the immune system. Modeling the cGAS-STING signaling pathway in distinct environments (infected or inflamed tissues, tumor, immune-privileged organs,…) and pathophysiological contexts (chronic inflammation, immunosuppression, …) constitutes an important challenge to improve the prediction of disease outcomes and reduce the high failure rates of clinical trials.

In this context, STING and cGAS KO mice have been instrumental for the advancement of knowledge and of drug discovery. However, different groups recently highlighted the limitations of such models. Oami and Coopersmith (135) discussed the fact that in these animal models, the gene is invalidated throughout the organism leading to strong phenotypes, which do not recapitulate the endogenous expression of cGAS-STING in various cell subtypes and tissues.

The design of cGAS-STING biosensors has been developed in parallel for example to detect and quantify the 2′-3′-cGAMP second messenger in mammalian cell extracts (136). The high sensitivity of such techniques allowed the measurement of 36 million molecules of 2′-3′-cGAMP produced on average per mammalian cell upon stimulation (136). Other strategies emerged to conduct high-throughput screening (HTS) or measure endogenous cGAMP using a STING-based biosensor (137) or a cGAMP-Luc reporter assay (138). Moreover, several commercial ELISA kits can be used to detect cGAMP in cells and tissues (139). These new tools are suitable for the discovery of cGAS-STING modulators although they are often studied in mouse models, while several studies report the species-specificity of STING ligand detection and activation (49, 140). Thus, further characterization of therapeutic compounds should be carried out with particular attention to the species specificities (6) of the cGAS-STING pathway and crosstalk mechanisms including the recently described STING-independent HSV-1 nucleic acid sensing (124, 141). High-throughput screenings of therapeutic molecules in zebrafish larvae can be considered as a promising approach since this biomedical model is suitable to study human inflammatory pathologies (AGS syndrome, cancer, and infectious diseases) (6, 142–144). Finally, organoids obtained from pluripotent stem cells from patients will soon constitute novels and complementary tools for considering personalized medicine (145). The drug repositioning strategy has also brought promising results (taking advantage of available clinical trials for toxicity and off-target side effects) while reducing the cost and development time of therapeutic candidates, as demonstrated by the interaction between antimalarial drugs and cGAS activities (103, 104). For instance, epigallocatechin gallate (EGCG) and aspirin were recently suggested as repurposed drugs inhibiting cGAS (146, 147).

Taken together, recent data on cGAS and STING structure and functions revealed the importance of this DNA sensing pathway in regulating the cellular response to pathogens as well as cell cycle and oncogenesis. Although additional studies would be required to get a comprehensive overview of the role of the cGAS platform in health and disease, the understanding of its molecular mode of action will pave the way to the development of urgently needed broad antiviral and anticancer strategies.

## Author Contributions

All authors listed have made a substantial, direct and intellectual contribution to the work, and approved it for publication.

## Conflict of Interest

The authors declare that the research was conducted in the absence of any commercial or financial relationships that could be construed as a potential conflict of interest.
